# Chemical Profiles of Incense Smoke Ingredients from Agarwood by Headspace Gas Chromatography-Tandem Mass Spectrometry

**DOI:** 10.3390/molecules23112969

**Published:** 2018-11-14

**Authors:** Wen-Yi Kao, Chien-Yun Hsiang, Shih-Ching Ho, Tin-Yun Ho, Kung-Ta Lee

**Affiliations:** 1 Department of Biochemical Science and Technology, National Taiwan University, Taipei 10617, Taiwan; mbkwi@dcb.org.tw; 2 Development Center for Biotechnology, New Taipei City 22180, Taiwan; scho@dcb.org.tw; 3 Department of Microbiology, China Medical University, Taichung 40402, Taiwan; cyhsiang@mail.cmu.edu.tw; 4 Graduate Institute of Chinese Medicine, China Medical University, Taichung 40402, Taiwan

**Keywords:** agarwood, headspace preheated system, gas chromatography-mass spectrometry, 2-(2-phenylethyl)chromone

## Abstract

Agarwood, the resinous wood in the heartwood of *Aquilaria* trees, has been used as incense in traditional Chinese medicine for its sedative, aphrodisiac, carminative, and anti-emetic effects. Grading of agarwood is usually based on its physical properties. Therefore, it is important to develop analytic methods for judgment and grading of agarwood. Here, we created a headspace (HS) preheating system that is combined with gas chromatography-mass spectrometry (HS GC-MS) to analyze the chemical constituents in the incense smoke produced by agarwood. Incense smoke generated in the HS preheating system was injected directly to GC-MS for analysis. A total of 40 compounds were identified in the incense smoke produced by Kynam agarwood, the best agarwood in the world. About half of the compounds are aromatics and sesquiterpenes. By analyzing chemical constituents in the incense smoke produced by Vietnamese, Lao, and Cambodian varieties of agarwood, we found that butyl hexadecanoate, butyl octadecanoate, bis(2-ethylhexyl) 1,2-benzenedicarboxylate, and squalene were common in the aforementioned four varieties of agarwoods. 2-(2-Phenylethyl) chromone derivatives were identified only in the incense smoke produced by Kynam agarwood, and were the major ingredient (27.23%) in the same. In conclusion, this is the first study that analyzes chemical profiles of incense smoke produced by agarwood using HS GC-MS. Our data showed that 2-(2-phenylethyl) chromone derivatives could be used to assess quality of agarwoods. Moreover, HS GC/MS may be a useful tool for grading quality of agarwood.

## 1. Introduction

Agarwood, also called Gaharu, Chén-xīang, Jin-koh, Kyara, or Oud, is the resinous wood formed in the heartwood of *Aquilaria* trees. *Aquilaria* species, including *A. sinensis* (Lour.) Glig., *A. agallocha* Roxb., and *A. malaccensis* Lam., taxonomically belong to Thymelaeaceae and are found mainly in Southeast Asia, such as Vietnam, Indonesia, Laos, Cambodia, and Malaysia [1,2]. Agarwood and its oil are important and useful natural substances that have been used to produce valuable products. Agarwood has been used as incense in religious ceremonies for centuries as well as in traditional Chinese medicine for its sedative, aphrodisiac, carminative, and anti-emetic effects [3,4,5,6,7,8,9]. The value of agarwood depends on its quality. However, grading of agarwood tends to be discretionary and subjective, because agarwood is usually graded based on its physical properties, such as color, density, resin formation, by sensory panels [1,2,10]. Kynam, also known as Kanankoh in Japan, is regarded to have the highest quality among different varieties of agarwood [11]. The high resin content, better water sinking quality, and darker color of Kynam enable it to produce a special and pleasant scent [1]. Kynam has been considered to be the noblest spiritual wood, both in ancient societies and in modern times, because of its scarcity. At present, the cost of Kynam agarwood is about 350 Euro per gram [12]. Kynam agarwood has a very complex scent, and even grading professionals cannot easily distinguish it from others. Therefore, it is important and essential to develop analytic methods for judgment and grading of agarwood.

Chemical constituents of agarwood have been studied by several research teams [1,3,13]. Previous studies identified chemical constituents of agarwood essential oils or organic solvent extracts with column chromatography or spectroscopic techniques [14,15]. Other studies prepared agarwood essential oils, hydrodistillates, or solvent extracts for gas chromatography (GC) or multidimensional GC analysis [16,17]. However, analysis of chemical constituents in agarwood solvent extracts is not suitable for differentiation of agarwood because agarwood is usually used to produce incense smoke. A few research studies analyzed chemical ingredients in the incense smoke produced by heated agarwood [11,18,19]. For example, Ishihara et al. [11] analyzed chemical constituents in the incense smoke that was trapped in the Tenax TA adsorbent resin, and extracted in diethyl ether for GC and GC-mass spectrometry (MS) analysis. They found 53 chemical compounds in the incense smoke produced by high quality (Kanankoh) and low quality (Jinkoh) varieties of Vietnamese agarwood, and most of them were sesquiterpenes and aromatics. Hung et al. [18] prepared an agarwood extract by heating a sealed vial that contained agarwood powder in a water bath at 90 °C for 30 min. The extract was absorbed in a solid-phase microextraction (SPME) needle with polydimethylsiloxane (PDMS) fiber for 30 min, and then desorbed for GC-MS analysis. They found six peaks that could be used for evaluating the quality or price of the most expensive agarwood. Zhou et al. [19] collected incense smoke produced by agarwood and incense sticks, absorbed the different kinds of collected incense smoke in glass fiber pads, and extracted substances from the glass fiber pads with dichloromethane for GC-MS analysis. Nevertheless, these studies analyzed chemical constituents in the incense smoke that was either absorbed in matrices or extracted in solvents.

Analysis of chemical constituents in the incense smoke produced by agarwood may be an approach to grading of agarwood. Most of past research studies determine chemical ingredients in agarwood solvent extracts [13,14,15,16,17]. However, analysis of chemical constituents in solvent extracts is not suitable in the case of agarwood because it is usually used to produce incense smoke. In this work, we used a headspace (HS) preheating system that is combined with GC-MS (HS GC-MS) to extract heavier sample matrices for GC analysis. Incense smoke produced by heated agarwood was directly analyzed with GC-MS. We could obtain more authentic chemical profiles of incense smoke produced by agarwood without solid-phase extraction. Moreover, by comparing chemical constituents in the incense smoke produced by different grades of agarwood, we found that HS GC/MS-MS could be used to grade quality of agarwood.

## 2. Results and Discussion

### 2.1. Morphological Observation of Various Grades of Agarwoods

**Figure 1:**
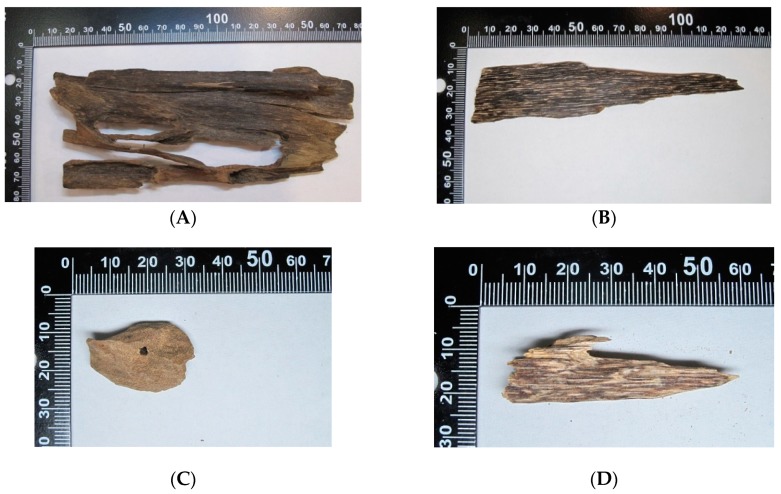
Morphological observation of Kynam (**A**), Vietnam (**B**), Liao (**C**), and Cambodian agarwoods (**D**).

Agarwood is traditionally graded based on its physical properties, such as resin content, water sinking quality, color, scent/aroma, as well as agarwood-inducing method, formation time, and place of origin [1,2]. Both Kynam and Vietnamese varieties of agarwood showed first-grade colors and had softer scents (Figure 1A,B). However, the Kynam variety fully sank in water, while the Vietnam variety merely partially submerged in water. Both the Lao and Cambodian varieties of agarwood fully floated up, showed second-grade colors, and had sweet scents (Figure 1C,D). However, the sweet scent of Cambodian agarwood was induced by insect infection.

### 2.2. HS GC-MS/MS Analysis

**Figure 2:**
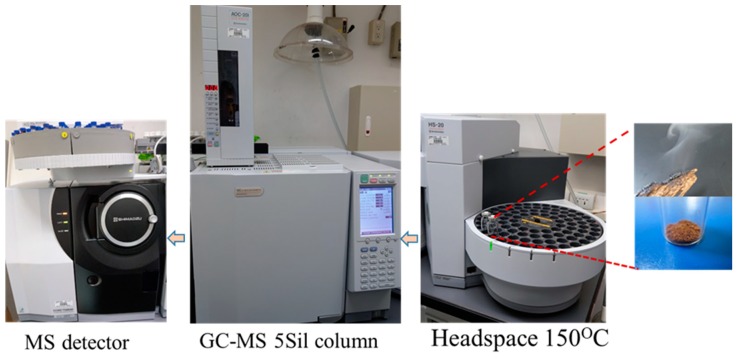
Experimental design and incense smoke detection process. Diagram shows that the incense smoke of agarwoods was generated by headspace preheated system and the smoke was injected directly to GC-MS for analysis.

Chemical constituents of agarwood have been intensively studied by several research teams [1,3,13]. Different extraction methods have been developed to determine chemical composition of essential oils and related compounds from agarwood chips using GC, GC-MS, solid phase microextraction, GC-flame ionization detector, GC-olfactometry, or comprehensive two-dimensional gas chromatography (GC×GC) [20]. Most studies suggest that hydrodistillation is the method of choice for determining the essential oil content of agarwood [7]. Agarwood has only a slight scent at room temperature, and releases a pleasant aroma when heated. Burning incense is a common traditional practice in many families and most temples in Asia, for religious reasons and for its pleasant smell [1,2]. Incense smoke contains many kinds of fragrant sesquiterpenes and aromatics [11]. Without interphase mass transfer, how to obtain a large amount of incense smoke is an important parameter for efficient analysis. Therefore, we prepared the incense smoke of agarwood using a HS preheating system, and tested the incubation times and temperatures to obtain the maximum amount of analytes. Previous studies extracted agarwood oils through incubation at 40 °C for 10 min, or at 90 °C for 30 min [18]. In this study, we prepared the incense smoke of agarwood through incubation at 150 °C for 30 min, and injected the smoke into GC-MS/MS for chemical profiling analysis (Figure 2).

### 2.3. Chemical Profiles of Kynam Agarwoods Using HS GC/MS-MS

**Figure 3:**
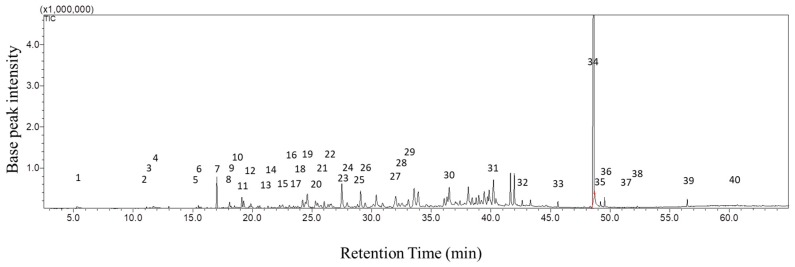
GC-MS chemical fingerprint of incense smoke from Kynam agarwood. Number indicates the identified compound, and the details of compounds are listed in Table 1.

**Figure 4:**
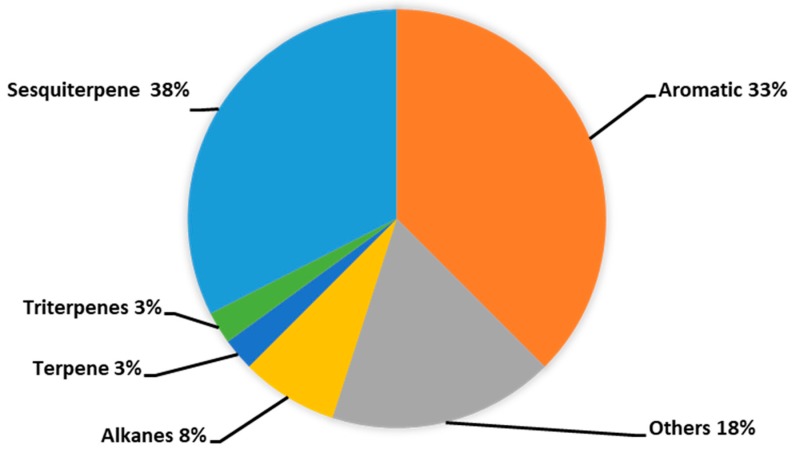
Pie chart showing chemical classification of 40 compounds identified in the incense smoke from Kynam agarwood.

Figure 3 and Figure S1 shows the GC chemical fingerprint of incense smoke produced by Kynam agarwood. The detection period was setup from 2.5 min to 65 min. Forty peaks were identified in the GC-MS/MS analysis, and the identified compounds are summarized in Table 1. As shown in Figure 4, these compounds belong to different kinds of chemical categories, including aromatics (33%), sesquiterpenes (38%), alkanes (8%), terpene (3%), triterpenes (3%), and others (18%). Since sesquiterpenes and triterpenes were mostly identified in agarwoods, other terpenes, except sesquiterpenes and triterpenes, were classified as “terpene” in this study. Moreover, the major component in the incense smoke of Kynam was 2-(2-phenylethyl) chromone (27.23%).

In 1993, Ishihara et al. [11] analyzed two kinds of agarwood incense smoke obtained with solvent extraction. Fifty components were identified in higher quality agarwood, while 36 components were identified in low quality agarwood. The incense smoke of both higher and low-quality agarwood was composed mainly of sesquiterpenes (53% and 61%, respectively) and aromatics (44% and 31%, respectively). The major components in higher quality agarwood were guaia-1(10), 11-dien-15-oic acid (9.92%), and 2-(2-phenylethyl) chromone (5.83%). Hung et al. [18] identified 17 components that could be used as markers for classification and differentiation of agarwood. 2-(2-Phenylethyl) chromone derivatives (flindersiachromone) were also identified in most expensive agarwoods.

A total of 132 constituents have been identified in solvent extracts from different varieties of agarwood in the past 5 decades, and they belong to sesquiterpenes (52%), 2-(2-phenylethyl) chromone derivatives (41%), and aromatics (1%) [3]. In this study, we found that aromatics and sesquiterpenes were the two major chemical categories in the incense smoke of Kynam agarwood as analyzed with HS GC-MS. The difference may result from the extraction procedure or varieties of agarwood.

Sesquiterpenes and 2-(2-phenylethyl) chromone derivatives were the two predominant constituents in agarwood [2]. As chromone derivatives could not be pyrolyzed [11], Espinoza et al. [20] used direct analysis with real time time-of-flight mass spectrometry (DART-TOFMS) to analyze 60 commercial agarwood chips without extraction, and identified the presence of key ions and the characteristics of 2-(2-phenylethyl) chromones. They found that 8–16 target chromone ions were present in each sample. These results showed that the highly oxidized agarwood chromones were specific to *Aquilaria* spp.

### 2.4. Chemical Profiles of Different Grades of Agarwoods Using HS GC/MS-MS

**Figure 5:**
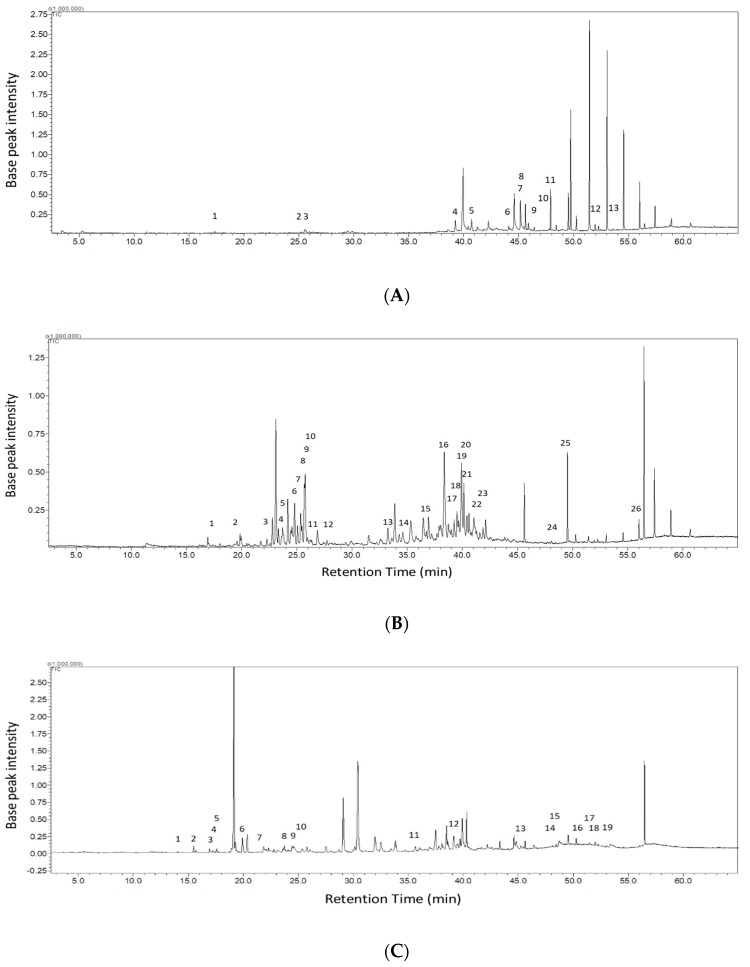

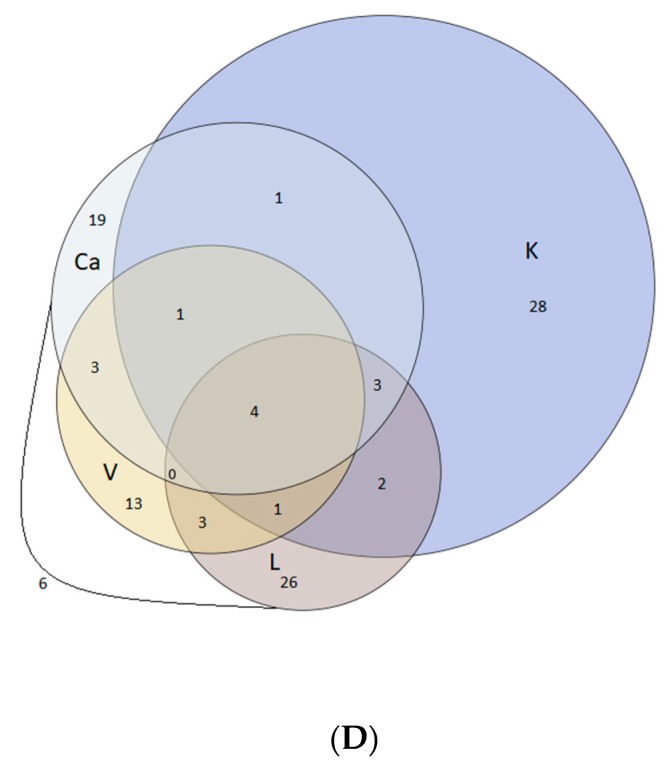
GC-MS chemical fingerprint of incense smoke from agarwoods. (**A**) Vietnam agarwood. (**B**) Liao agarwood. (**C**) Cambodian agarwood. Number indicates the compound specifically present in the agarwood. (**D**) Venn diagram of 110 compounds identified in the incense smoke from Kynam (K), Vietnam (V), Liao (L), and Cambodian (Ca) agarwoods.

To further compare chemical constituents in the incense smoke produced by Vietnamese, Lao, and Cambodian varieties of agarwood with those in the same produced by the Kynam variety, we prepared the incense smoke using a HS preheating system, and the smoke was directly injected to GC for chemical identification. Lao agarwood shares similar physical properties with Cambodian agarwood, and both varieties exhibited similar patterns in GC (Figure 5 and Figure S2). A total of 110 constituents were identified in the incense smoke produced by four varieties of agarwood, including 40 compounds in the Kynam variety, 25 compounds in the Vietnamese variety, 45 compounds in the Lao variety, and 31 compounds in the Cambodian variety. Four compounds were commonly identified in the four varieties of agarwood, while 28, 13, 26, and 19 compounds were specifically identified in the Kynam, Vietnamese, Lao, and Cambodian varieties of agarwood, respectively (Figure 5D and Table 2). Butyl hexadecanoate, butyl octadecanoate, bis(2-ethylhexyl) 1,2-benzenedicarboxylate, and aqualene were commonly found in the incense smoke produced by the four varieties of agarwood. Ishihara et al. [11] analyzed the smoke profiles of two different varieties of Vietnamese agarwood that were absorbed in Tenax TA, and found small amounts of benzaldehyde and 2-(2-phenylethyl) chromone were present in both varieties of Kynam (Kanakoh) agarwood. Ismail et al. [21] analyzed the chemical constituents of agarwood oil that was absorbed in PDMS and divinylbenzene-carboxen-PDMS, and found that 4-phenyl-2-butanone was one of the major compounds that contributed to the scent. 4-Methoxy-benzaldehyde was the first compound identified in the incense smoke of agarwood in this study, whereas it had not been identified in agarwood smoke or oils in other studies. These findings indicate that the identification of constituents in agarwood is affected by adsorbents. Therefore, more authentic chemical profiles of agarwood can be achieved with the use of incense smoke without matrix absorption.

### 2.5. Chemical Ingredients of Different Grades of Agarwoods

Sesquiterpenoids and 2-(2-phenylethyl) chromone derivatives are two predominant constituents in agarwood [1,2,3]. Sesquiterpenoids, the essential ingredients in luxury perfumes, have been identified in different varieties of agarwood. In addition to sesquiterpenes, 2-(2-phenylethyl) chromone derivative (flindersiachromone, 2-phenethyl-4*H*-chromen-4-one) is another key reference constituent in agarwood. 2-(2-Phenylethyl)chromone derivatives can only be extracted in solvents or supercritical CO_2_, and are never found in hydrodistillates [22,23,24]. Substitute chromones in different grades of agarwood have also been studied. 2-(2-Phenylethyl) chromone derivatives can be detected in 76% of agarwood chips using direct analysis with DART-TOFMS without chemical extraction; however, DART-TOFMS is only used for distinguishing *Aquilaria* genus from others instead of agarwood grading [20]. One previous study showed that 2-(2-phenylethyl)chromone derivatives were found in incense smoke produced by Vietnamese agarwood, with a GC peak area of 5.83% in Kanankoh (high quality variety of agarwood) [11]. Another study also showed that 2-(2-phenylethyl)chromone derivatives were present only in expensive agarwood [15]. In this study, we found that 2-(2-phenylethyl) chromones were identified in the incense smoke of the Kynam variety (27.23%), but not in the same of Vietnamese, Lao, or Cambodian variety. So far, 39 different 2-(2-phenylethyl) chromone derivatives have been identified in different varieties of agarwood [20,25]. In line with these studies, our findings suggest that analysis of 2-(2-phenylethyl) chromone derivatives can be used to assess quality of agarwood. Moreover, the contents of 2-(2-phenylethyl) chromone derivative in triple duplicates of Kynam smoke were 25.18%, 27.23%, and 26.17% (Supplementary Materials Figure S1). Therefore, our data suggested that the HS GC/MS developed in this study can be a useful tool for grading quality of agarwood through direct analysis of incense smoke.

## 3. Materials and Methods

### 3.1. Reagents

All chemicals were purchased from Sigma (St. Louis, MO, USA), unless indicated.

### 3.2. Agarwood Materials

All natural agarwoods, including Kynam, Vietnam, Liao and Cambodia, were purchased from Chenglin Inc. (Yunlin, Taiwan). Agarwoods used in this study were identified and qualified by The International Tropical Timber Organization. The voucher specimen has been deposited in Development Center for Biotechnology. The agarwood chips were pulverized into a fine powder before use.

### 3.3. Sample Preparation

The incense smoke of agarwood was prepared using HS preheated system. Agarwood powder (50 mg) was weighted and placed in a 20 mL glass vial. The vial was sealed with Teflon, shaken, and incubated at 150 °C for 30 min in a headspace autosampler oven (Model HS-20, Shimadzu Corporation, Kyoto, Japan).

### 3.4. GC-MS/MS Analysis

Chemical constituents of incense smoke were analyzed by GS-MS/MS. Incense smoke (1 mL) was injected into GC-MS/MS (Model GC-2010 Plus and GCMS-TQ8040, Shimadzu Corporation, Kyoto, Japan). Temperature of sample line and transfer line was kept at 150 °C Ultra-high purity helium (99.99%) was used as the carrier gas at a flow rate of 5.1 mL/min. The chromatographic separation was conducted with SH-Rxi-5Sil MS capillary column (30 m × 0.25 mm inner diameter, 0.25 μm thickness, Shimadzu Corporation, Kyoto, Japan). The column temperature program was set as follows: 60 °C initial temperature and subsequently ramped to 140 °C at a rate of 6 °C/min, held at 140 °C for 5 min, ramped to 160 °C at a rate of 2 °C/min, held at 160 °C for 5 min, ramped to 280 °C at a rate of 5 °C/min, held at 280 °C for 10 min. The GC-MS interface temperature was maintained at 250 °C. Electron ionization was used as the ionization method, and the ion source temperature was set at 230 °C. The mass spectra obtained with full scan and the mass ranged from *m*/*z* 45 to 550 were used for determining the unknown components.

Raw data were processed using GCMS solution Version 4.41 (Shimadzu Corporation, Kyoto, Japan). Mass spectral fragmentation patterns were compared with those stored in the NIST Mass Spectral Library (nist14), which is built up by using the pure substances and the mass spectra from literatures. In order to get the linear retention index values of the volatile compounds, a series of *n*-alkanes calibration standard (C8–C40) was run in the same condition (Figure S3). A Venn diagram was generated by eulerr program of R package [26].

## 4. Conclusions

So far, grading of agarwoods is flexible and subjective, because of the absence of standard grading system. Scent is the most important parameter for the quality assessment of agarwoods. In this work, we established a simple, automated, robust, and high-throughput method to analyze the incense smoke generated from a minimum amount of agarwood sample by HS GC-MS/MS. In comparison with the incense smoke constituents in Vietnam, Liao, and Cambodian agarwoods, we found that Kynam agarwood was composed of more complex constituents. Moreover, 2-(2-phenylethyl)chromone derivative was present in Kynam instead of Vietnam, Liao, and Cambodia agarwoods, suggesting that the content of 2-(2-phenylethyl)chromone derivative could be applied to grade the quality of agarwoods.

## Figures and Tables

**Table 1:** List of compounds identified in the incense smoke from Kynam agarwood by GC-MS/MS.

No.	Compound	LRI ^a^	Retention Time (min)	Content ^b^ (%)	Detected *m*/*z*	Chemical Formula	Type ^c^
1	Benzaldehyde	1581	5.296	0.17 ± 0.05	106	C_7_H_6_O	Ar
2	exo-7-(2-Propenyl)bicyclo [4.2.0]oct-1(2)-ene	1720	11.137	0.04 ± 0.02	148	C_11_H_16_	Other
3	4-Methoxybenzaldehyde	1731	11.617	0.14 ± 0.05	136	C_8_H_8_O_2_	Ar
4	1,8-Nonadiene-3-yne, 2,8-dimethyl-7-methylene-	1734	11.717	0.04 ± 0.03	160	C_12_H_16_	Other
5	Cubenene	1818	15.273	0.03 ± 0.02	204	C_15_H_24_	S
6	α-Longipinene	1823	15.471	0.26 ± 0.12	204	C_15_H_24_	S
7	Tricyclo[5 2.2.0(1,6)]undecan-3-ol, 2-methylene-6,8,8-trimethyl-	1860	17.017	1.30 ± 0.42	220	C_15_H_24_O	Ar
8	Naphthalene	1885	18.086	0.18 ± 0.22	202	C_15_H_22_	Ar
9	δ-Guaiene	1888	18.196	0.13 ± 0.08	204	C_15_H_24_	S
10	Nootkatene	1895	18.502	0.12 ± 0.06	202	C_15_H_22_	Ar
11	Kessane	1910	19.119	0.39 ± 0.29	222	C_15_H_26_O	Ar
12	4a,5-Dimethyl-3-(prop-1-en-2-yl)-1,2,3,4,4a,5,6,7-octahydronaphthalen-1-ol	1928	19.863	0.31 ± 0.07	220	C_15_H_24_O	Ar
13	*cis*-α-Santalol	1962	21.312	0.28 ± 0.09	220	C_15_H_24_O	S
14	β-Vetivenene	1969	21.593	0.10 ± 0.02	202	C_15_H_22_	T
15	γ-Eudesmol	1998	22.799	0.06 ± 0.06	222	C_15_H_26_O	S
16	10s,11s-Himachala-3(12),4-diene	2017	23.61	0.27 ± 0.12	204	C_15_H_24_	Ar
17	Agarospirol	2022	23.808	0.21 ± 0.04	222	C_15_H_26_O	S
18	β-Guaiene	2031	24.209	0.53 ± 0.24	204	C_15_H_24_	S
19	Cubenol	2041	24.606	1.24 ± 0.43	222	C_15_H_26_O	S
20	α-epi-7-epi-5-Eudesmol	2057	25.296	0.95 ± 0.28	222	C_15_H_26_O	S
21	Bulnesol	2074	26.021	0.26 ± 0.18	222	C_15_H_26_O	S
22	α-Tetralone	2089	26.624	0.53 ± 0.26	218	C_15_H_22_O	Ar
23	α-Kessyl acetate	2110	27.497	0.91 ± 1.31	280	C_17_H_28_O_3_	Ar
24	(1*R*,7*S*,*E*)-7-Isopropyl-4,10-dimethylenecyclodec-5-enol	2120	27.941	0.33 ± 0.32	220	C_15_H_24_O	Ar
25	10-epi-Elemol	2141	28.822	0.19 ± 0.15	222	C_15_H_26_O	S
26	Ledol	2156	29.445	0.53 ± 0.10	222	C_15_H_26_O	S
27	Longiverbenone	2217	32.017	0.82 ± 0.74	218	C_15_H_22_O	Other
28	Cryptomeridiol	2230	32.552	1.95 ± 1.10	240	C_15_H_28_O_2_	S
29	2a*S*,3a*R*,5a*S*,9b*R*)-2a,5a,9-Trimethyl-2a,4,5,5a,6,7,8,9b-octahydro-2*H*-naphtho[1–b]oxireno[2–c]furan	2243	33.081	0.40 ± 0.45	234	C_15_H_22_O_2_	Ar
30	10-*epi*-γ-Eudesmol	2324	36.506	2.21 ± 0.19	222	C_15_H_26_O	S
31	Sandaracopimarinal	2413	40.221	2.19 ± 0.57	286	C_20_H_30_O	Ar
32	γ-Gurjunene	2470	42.629	0.52 ± 0.10	204	C_15_H_24_	S
33	Butyl hexadecanoate	2542	45.628	0.75 ± 0.46	312	C_20_H_40_O_2_	Other
34	2-(2-Phenylethyl)chromone	2613	48.64	26.19 ± 1.0	250	C_17_H_14_O_2_	Other
35	Butyl octadecanoate	2635	49.538	1.13 ± 0.69	340	C_22_H_44_O_2_	Other
36	1-Iodo-octacosane	2640	49.743	0.05 ± 0.03	520	C_28_H_61_I	Ak
37	1-Iodo-eicosane	2680	51.447	0.09 ± 0.06	408	C_20_H_41_I	Ak
38	Bis(2-ethylhexyl) 1,2-benzenedicarboxylate	2700	52.277	0.34 ± 0.38	390	C_24_H_38_O_4_	Other
39	Squalene	2806	56.472	1.19 ± 1.30	410	C_30_H_50_	Tri
40	Nonacosane	2900	60.669	0.08 ± 0.05	408	C_29_H_60_	Ak

^a^ LRI: Linear retention index as tested on SH-Rxi-5Sil column using the series of C8–40 *n*-alkanes; ^b^ Values are the mean ± standard error (*n* = 3); ^c^ Ak: alkane; Ar: aromatics; S: sesquiterpene; T: terpene; Tri: triterpene.

**Table 2:** Compounds commonly or specifically present in the incense smoke of agarwoods.

Compound	Retention Time (min)	LRI	Detected *m*/*z*	Chemical Formula	Agarwoods ^a^
Butyl hexadecanoate	45.628	2542	312	C_17_H_40_O_2_	K, V, L, C
Butyl octadecanoate	49.538	2635	340	C_22_H_44_O_2_	K, V, L, C
Bis(2-ethylhexyl) 1,2-benzenedicarboxylate	52.277	2700	390	C_24_H_38_O_4_	K, V, L, C
Squalene	56.472	2800	410	C_30_H_50_	K, V, L, C
Benzaldehyde	5.296	1581	106	C_7_H_6_O	K
4-methoxy-Benzaldehyde	11.617	1731	136	C_8_H_8_O_2_	K
1,8-Nonadien-3-yne, 2,8-dimethyl-7-methylene-	11.717	1734	160	C_12_H_16_	K
Cubenene	15.275	1818	204	C_15_H_24_	K
α-Longipinene	15.471	1823	204	C_15_H_24_	K
Tricyclo[5.2.2.0(1,6)]undecan-3-ol, 2-methylene-6,8,8-trimethyl-	17.017	1860	220	C_15_H_24_O	K
Naphthalene	18.086	1885	202	C_15_H_22_	K
δ-Guaiene	18.196	1888	204	C_15_H_24_	K
Nootkatene	18.502	1895	202	C_15_H_22_	K
4a,5-Dimethyl-3-(prop-1-en-2-yl)-1,2,3,4,4a,5,6,7-octahydronaphthalen-1-ol	19.863	1928	220	C_15_H_24_O	K
*cis*-α-Santalol	21.312	1962	220	C_15_H_24_O	K
β-Vetivenene	21.593	1969	202	C_15_H_22_	K
γ-Eudesmol	22.799	1998	222	C_15_H_26_O	K
10s,11s-Himachala-3(12),4-diene	23.610	2017	204	C_15_H_24_	K
β-Guaiene	24.209	2031	204	C_15_H_24_	K
Cubenol	24.606	2041	222	C_15_H_26_O	K
α-Tetralone	26.624	2089	218	C_15_H_22_O	K
α-Kessyl acetate	27.497	2110	280	C_17_H_28_O_3_	K
(1*R*,7*S*,*E*)-7-Isopropyl-4,10-dimethylenecyclodec-5-enol	27.941	2120	220	C_15_H_24_O	K
Ledol	29.445	2156	222	C_15_H_26_O	K
Longiverbenone	32.017	2217	218	C_15_H_22_O	K
2a*S*,3a*R*,5a*S*,9b*R*)-2a,5a,9-Trimethyl-2a,4,5,5a,6,7,8,9b-octahydro-2*H*-naphtho[1–b]oxireno[2–c]furan	33.081	2243	234	C_15_H_22_O_2_	K
10-*epi*-γ-Eudesmol	36.506	2324	222	C_15_H_26_O	K
Sandaracopimarinal	40.221	2413	286	C_20_H_30_O	K
γ-Gurjunene	42.629	2470	204	C_15_H_24_	K
2-(2-Phenylethyl)chromone	48.640	2613	250	C_17_H_14_O_2_	K
1-iodo-Triacontane	49.743	2640	548	C_22_H_44_O_2_	K
Eicosane, 1-iodo-	51.447	2680	408	C_20_H_41_I	K
2-Isopropenyl-4a,8-dimethyl-1,2,3,4,4a,5,6,7-octahydronaphthalene	17.354	1868	204	C_15_H_24_	L
Dehydrodeoxybaimuxinol	19.844	1927	220	C_15_H_24_O	L
Rosifoliol	22.267	1985	222	C_15_H_26_O	L
1H-Cyclopropa[a]naphthalene, 1a,2,3,3a,4,5,6,7b-octahydro-1,1,3a,7-tetramethyl-, [1aR-(1aα,3aα,7bα)]-	23.598	2017	204	C_15_H_24_	L
Guaiol acetate	23.692	2019	222	C_15_H_26_O	L
Isovalencenol	24.782	2045	218	C_15_H_22_O	L
4-isopropenyl-1-methoxymethoxymethyl-cyclohexene	25.046	2051	196	C_12_H_20_O_2_	L
*trans*-α-Bisabolene	25.447	2061	204	C_15_H_24_	L
β-Patchoulene	25.667	2066	204	C_15_H_24_	L
Androstan-17-one, 3-ethyl-3-hydroxy-, (5α)-	25.938	2072	318	C_21_H_34_O_2_	L
Acetate, (2,4a,5,8a-tetramethyl-1,2,3,4,4a,7,8,8a-octahydro-1-naphthalenyl) ester	26.337	2082	250	C_16_H_26_O_3_	L
Dehydrofukinone	27.707	2115	218	C_15_H_22_O	L
Neoisolongifolene, 8,9-epoxy-	33.235	2246	218	C_15_H_22_O	L
6-Isopropenyl-4,8a-dimethyl-1,2,3,5,6,7,8,8a-octahydro-naphthalen-2-ol	34.576	2278	220	C_15_H_24_O	L
(*S*)-*cis*-Verbenol	36.717	2329	152	C_10_H_16_O	L
Card-20(22)-enolide, 3,5,14,19-tetrahydroxy-, (3β,5β)-	38.348	2368	406	C_23_H_34_O_6_	L
Espatulenol	39.248	2390	220	C_15_H_24_O	L
5-Isopropenyl-2-methylcyclopent-1-enecarboxaldehyde	39.504	2396	150	C_10_H_14_O	L
Levoverbenone	39.650	2399	150	C_10_H_14_O	L
Cyclobutene, 4,4-dimethyl-1-(2,7-octadienyl)-	39.908	2405	190	C_14_H_22_	L
Alloaromadendrene oxide-(1)	40.407	2417	220	C_15_H_24_O	L
3-Oxatricyclo[20.8.0.0(7,16)]triaconta-1(22),7(16),9,13,23,29-hexaene	41.858	2452	406	C_29_H_42_O	L
Andrographolide	42.100	2458	350	C_20_H_30_O_5_	L
Docosa-2,6,10,14,18-pentaen-22-al, 2,6,10,15,18-pentamethyl-, all-trans	48.062	2600	384	C_27_H_44_O	L
2,6,10-trimethyl-Tetradecane	49.731	2639	240	C_17_H_36_	L
Heptadecanal	51.965	2693	254	C_17_H_34_O	L
4,5-di-epi-Aristolochene	17.343	1868	204	C_15_H_24_	V
α-Costal	25.156	2054	218	C_15_H_22_O	V
6,7-Dimethyl-1,2,3,5,8,8a-hexahydronaphthalene	25.964	2073	162	C_12_H_18_	V
Dihydrocolumellarin	39.222	2389	234	C_15_H_22_O_2_	V
Columellarin	40.694	2424	232	C_15_H_20_O_2_	V
Isovelleral	44.081	2505	232	C_15_H_20_O_2_	V
Octadecanoic acid	45.149	2530	284	C_18_H_36_O_2_	V
1-Hexacosene	45.755	2545	364	C_26_H_52_	V
Pentadecanal-	46.405	2560	226	C_15_H_30_O	V
*n*-Pentadecanol	47.783	2593	228	C_15_H_32_O	V
2-Nonadecanone	48.071	2600	282	C_19_H_38_O	V
Dotriacontane, 1-iodo-	52.442	2704	576	C_32_H_65_I	V
Tetracosanal	53.568	2731	352	C_24_H_48_O	V
α-Ylangene	14.047	1789	204	C_15_H_24_	C
5,5-dimethyl-4-(3-oxobutyl)-Spiro[2.5]octane	15.489	1824	208	C_14_H_24_O	C
Diepicedrene-1-oxide	17.013	1860	220	C_15_H_24_O	C
7-*epi*-α-Cadinene	17.220	1865	204	C_15_H_24_	C
α-Guaiene	17.385	1869	204	C_15_H_24_	C
Alloaromadendrene oxide-(2)	19.874	1928	220	C_15_H_24_O	C
Diethyl phthalate	21.888	1976	222	C_12_H_14_O_4_	C
Hinesol	23.982	2026	222	C_15_H_26_O	C
*cis*-Eudesm-6-en-11-ol	24.456	2037	222	C_15_H_26_O	C
Longipinane, (*E*)-	25.371	2059	206	C_15_H_26_	C
Pentadecanoic acid	35.990	2312	242	C_15_H_30_O_2_	C
Palmitoleic acid	39.168	2388	254	C_16_H_30_O_2_	C
Hexadecane	45.448	2537	226	C_16_H_34_	C
*trans*-Geranylgeraniol	48.077	2600	290	C_20_H_34_O	C
Tetradecanal	48.432	2608	212	C_14_H_28_O	C
Hexadecanal	50.261	2652	240	C_16_H_32_O	C
Octacosane	51.439	2680	394	C_28_H_58_	C
Oxirane, hexadecyl-	51.970	2693	268	C_18_H_36_O	C
Heptacosane	53.037	2718	380	C_27_H_56_	C

^a^ K: Kynam; V: Vietnam; L: Liao; C: Cambodian.

## Supplementary Materials

The Supplementary Materials are available online.
