# Supplementary material for: Chemical Profiles of Incense Smoke Ingredients from Agarwood by Headspace Gas Chromatography-Tandem Mass Spectrometry
Source: Molecules. 2018 Nov 14;23(11):2969. doi: 10.3390/molecules23112969 (PMC6278519; doi:10.3390/molecules23112969)
Supplement: Supplementary file 1 [file molecules-23-02969-s001.pdf]

Chemical Profiles of Incense Smoke Ingredients from Agarwood by Headspace Gas Chromatography - Tandem Mass Spectrometry

Wen-Yi Kao, Chien-Yun Hsiang, Shih-Ching Ho, Tin-Yun Ho, and Kung-Ta Lee

Supplementary materials

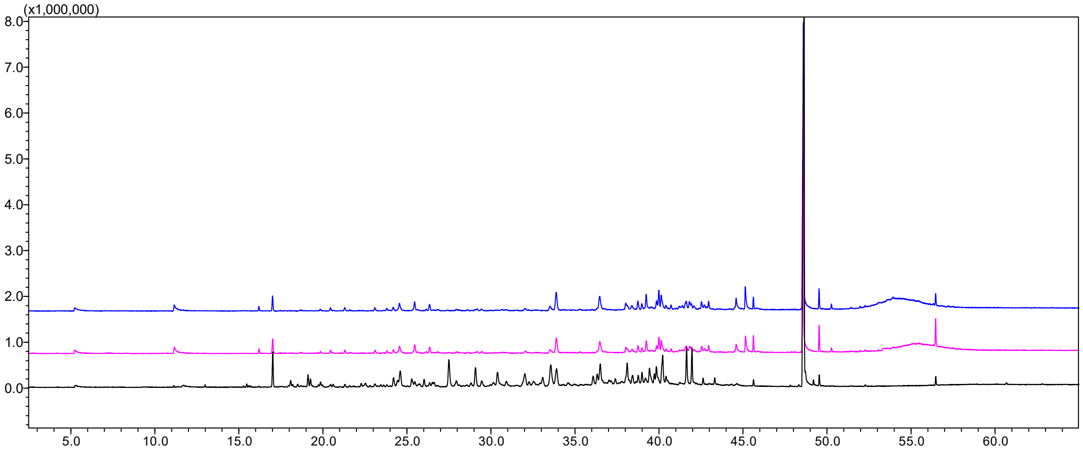

Figure S1. GC chemical fingerprint of incense smoke from Kynam agarwood. GC analysis was performed in triplicate.

(A)

(B)

(C)

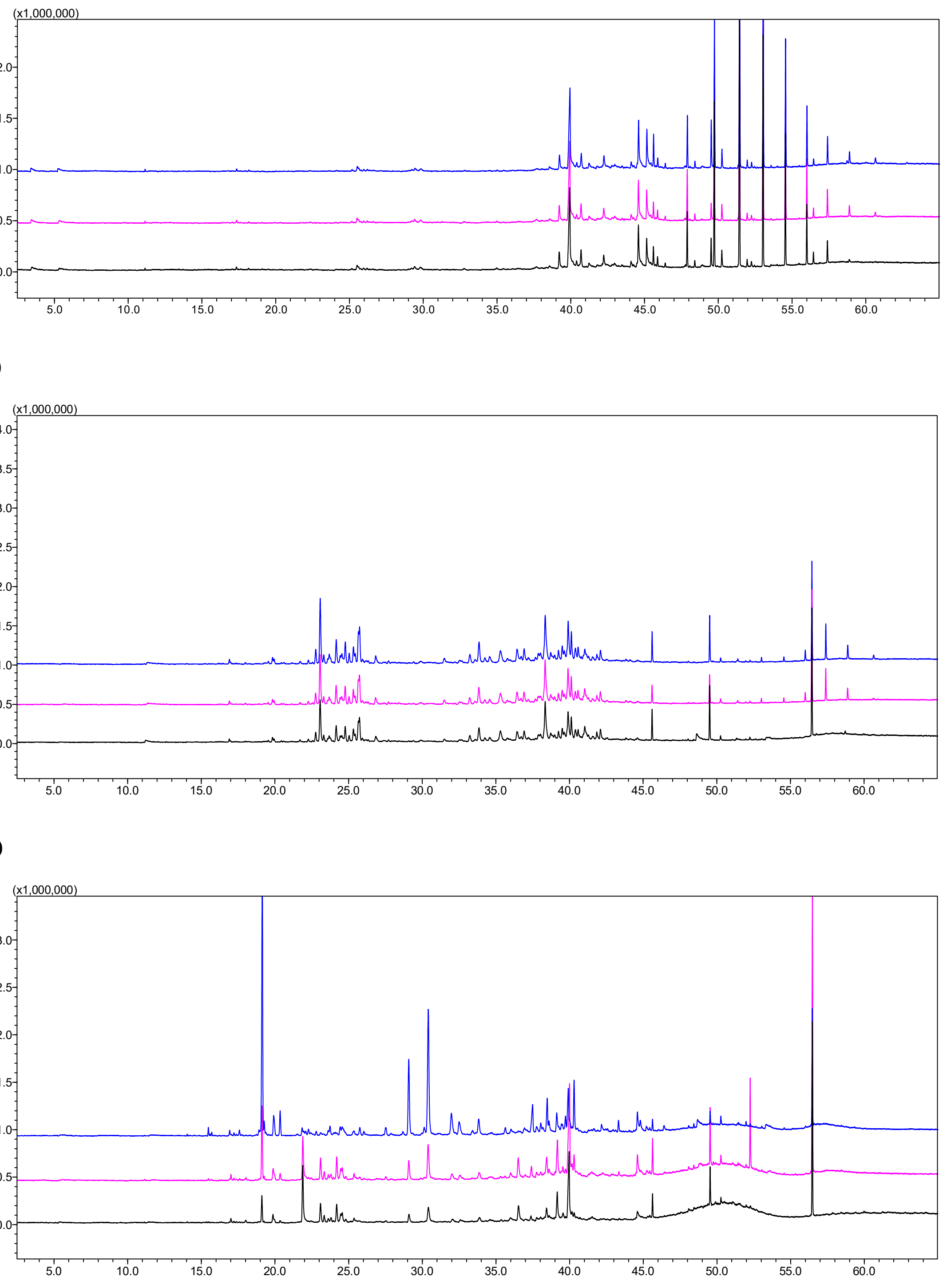

Figure S2. GC chemical fingerprint of incense smoke from Vietnam (A), Liao (B), and Cambodia agarwoods (C). GC analysis was performed in triplicate.

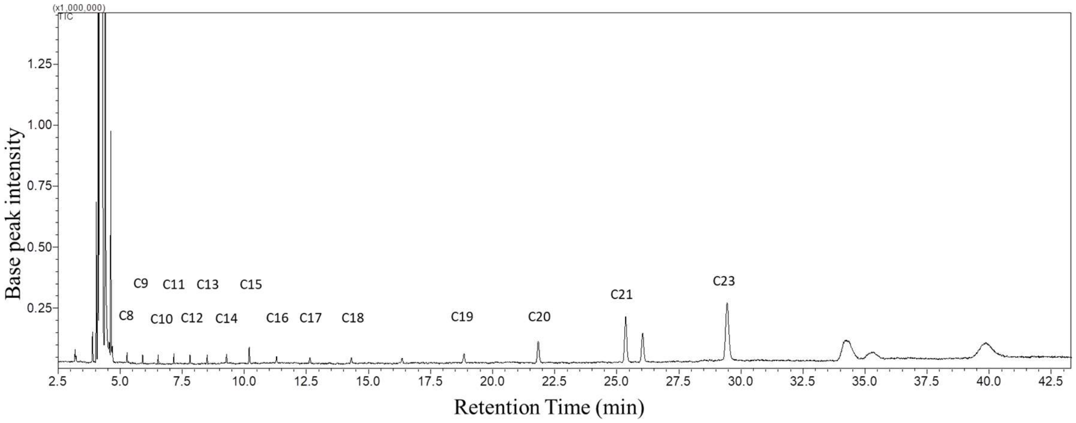

Figure S3. GC chromatogram of n-alkanes calibration standard (C8-C40).
